# Maprotiline ameliorates isoflurane-induced microglial activation via regulating triggering receptor expressed in myeloid cells 2 (TREM2)

**DOI:** 10.1080/21655979.2021.2000740

**Published:** 2021-12-11

**Authors:** Rui Hu, Yongguan He, Zhigang Chen

**Affiliations:** a Department of Anesthesiology, Affiliated Hospital of Guilin Medical College, Guilin, China; b Department of Anesthesiology, The Central Hospital of Enshi Tujia and Miao Autonomous Prefectrue, Enshi Tujia and Miao Autonomous Prefecture, Hubei, China; c Department of Anesthesia and Pain, wuhanxinzhou District People’s Hospital, Wuhan, China

**Keywords:** Isoflurane, maprotiline, microglial activation, oxidative stress, inflammation, TREM2

## Abstract

Isoflurane-induced neurotoxicity has attracted much interest. Recent studies suggest that isoflurane causes microglial activation, resulting in an inflammatory response and microglial insult. Maprotiline is a novel drug that has been licensed as an antidepressant with considerable anti-inflammatory activity. However, it is still unknown whether maprotiline possesses a protective effect against isoflurane-induced microglial insult. Here, we found that maprotiline ameliorated isoflurane-caused reduction in BV2 microglial cell viability and lactate dehydrogenase (LDH) release. Maprotiline mitigated isoflurane-induced oxidative stress by inhibiting reactive oxygen species (ROS) production and increasing superoxide dismutase (SOD) activity. Isoflurane-induced expression and production of inflammatory markers including tumor necrosis factor (TNF-α), interleukin (IL)-1β, cyclooxygenase‐2 (COX-2), and prostaglandin E2 (PGE_2_) were decreased in maprotiline-treated cells. Maprotiline inhibited the mRNA and protein levels of Iba1, a marker of microglial activation, in isoflurane-induced BV2 cells. Maprotiline treatment restored isoflurane-induced reduction of TREM2 in BV2 microglial cells. In addition, the knockdown of TREM2 abolished the beneficial effects of maprotiline against isoflurane. Collectively, maprotiline exerted protective effects against isoflurane-caused oxidative stress, inflammatory response, and cell injury via regulating TREM2. These findings show that maprotiline prevented the isoflurane-induced microglial activation, indicating that maprotiline might be used as an optimal therapeutic agent for preventing the isoflurane-caused neurotoxicity.

## 1. Introduction

Isoflurane (C_3_H_2_ClF_5_O) is one of the most clinically used volatile anesthetic agents in surgical procedures [1]. Currently, its effects on brain tissue are inconsistent. Although it has been found to possess neuroprotective effects in adult patients, it also has a neurotoxic effect in newborn and elderly patients [2]. Isoflurane may affect the recovery and neurological capabilities of newborn and elderly patients. Studies regarding the neurotoxic effect of isoflurane prove that it exhibits neuro-apoptotic effects with increased caspase-3 activity, elevated Bax level, and reduced Bcl-2 level in aged rats [3]. Isoflurane induces cognitive impairment in aged mouse models by inducing the production of brain cytokines including TNF-α, IL-1β, and IL-6 via suppressing the mammalian target of rapamycin (mTOR) signaling [4,5]. Isoflurane also leads to increased production of reactive oxygen species (ROS), disruption in the mitochondrial membrane, oxidative stress, and cell death in primary neuronal cultures [6].

Microglia act as the innate immune effector of the brain and are implicated in several functions: regulation of inflammation, programmed cell death, synaptic connectivity, and phagocytosis [7]. Alterations in the status of microglial activation switch microglia to an inflammation phenotype with loss of neuroprotective functions, which may lead to neurodegenerative diseases [8]. There is evidence that isoflurane may cause activation of microglia, resulting in an inflammatory response and oxidative stress, and ultimately to microglial insult [9–11]. Therefore, a definitive characterization of isoflurane toxicity and effective clinical intervention is crucial for its safe handling in the clinic.

Maprotiline is a tetracyclic drug with sedative and anti-aggressive properties in animals. It is used for the treatment of mental depression and depressive mood disorders [12]. Recent pharmacological studies have shown that maprotiline has strong anti-inflammatory activity. It inhibits inflammatory gene expression in lipopolysaccharide (LPS)-stimulated macrophages and carrageenan injection-caused paw edema in rats [13]. Maprotiline attenuates the inflammatory response in edema by regulating the migration of neutrophils and degranulation of mast cells. However, whether maprotiline has the capacity to improve isoflurane-caused neuroinflammation in microglia remains unknown. Here, we studied the effects of maprotiline on isoflurane-induced cell damage in BV2 microglial cells.

## 2. Materials and methods

### 2.1 BV2 cells culture and transduction

Before cell transfection, BV2 cells were grown in high glucose Dulbecco’s Modified Eagle Medium (DMEM) (Hyclone, Logan, UT, USA), supplemented with 5% fetal bovine serum (FBS) (Hyclone) and maintained at 37°C in 5% CO_2_ incubator.

For the cell transduction, the BV2 cells were replated at the density of 5 × 10^4^ cells/well in a 24-well plate. The lentiviral-triggering receptor expressed on myeloid cells 2 (TREM2) shRNA (LV-shTREM2) and negative control lentiviral-NC shRNA (LV-shNC) (GeneChem, Shanghai, China) were added to the BV2 cells for 48 h. Then, the cell pellets were harvested and the TREM2 protein level was detected using western blotting.

### 2.2 WST-1 assay

For determining the cell viability of BV2 cells, the WST-1 assay was performed using a kit (Roche Diagnostics, Mannheim, Germany). BV2 cells were incubated with 110 μL of working solution for 2 h, and the absorbance was measured at 450 nm.

### 2.3 Release of lactate dehydrogenase (LDH)

For determining the cytotoxicity, 100 μL of BV2 cells supernatant was collected for LDH release assay using a kit (Clontech Laboratories, Mountain View, CA). The samples were incubated with 100 μL of the reaction mixture for 30 min. Absorbance was then measured at 490 nm.

### 2.4 MitoSOX red staining

The level of mitochondrial ROS was evaluated using MitoSox‐Red, which is an indicator of mitochondrial superoxide. After different treatments, BV2 cells were probed with 5 μmol/L MitoSox‐Red (Life Technologies, Grand Island, NY) for 30 min in the dark at 37°C. Afterward, BV2 cells were washed and subjected to a fluorescent microscope for the analysis of red fluorescence [14].

### 2.5 Superoxide dismutase (SOD) activity

The activity of SOD was measured with nitro blue tetrazolium (NBT) reaction method using a commercial kit (Beyotime Bio, Nantong, China). Finally, the color reaction was measured at 560 nm.

### 2.6 Real-time (RT)-PCR

After the indicated treatments, the total RNA of BV2 cells was extracted using TRIzol (Invitrogen) and then reverse-transcribed into cDNAs using a reverse transcription kit (TaKaRa Bio, Japan). RT-PCR was subsequently performed for the determination of TNF-α, IL-1β, COX-2, Iba1, and TREM2 using Prime Taq Premix (TaKaRa Bio). The 2^−ΔΔCt^ method was used to calculate the expression of target genes [15]. The following primers were used in the study: TNF-α (forward: 5ʹ- GACGTGGAACTGGCAGAAGA-3ʹ, reverse: 5ʹ-GGCTACAGGCTTGTCACTCG-3ʹ); IL-1β (forward: 5ʹ-TCGCAGCAGCACATCAACAAGAG-3ʹ, reverse: 5ʹ- TGCTCATGTCCTCATCCTGGAAGG-3ʹ); COX-2 (forward: 5ʹ-CCTTCCTCCTGTGCCTGATG-3ʹ, reverse: 5ʹ-ACAATCTCATTTGAATCAGGAAGCT-3ʹ); TREM2 (forward: 5ʹ- TCAGGGAGTCAGTCATTAACCA-3ʹ, reverse: 5ʹ-AGTGCTTCAAGGCGTCATAAGT-3ʹ); β-actin (forward: 5ʹ-GGTCATCACTATTGGCAACG-3ʹ; reverse: 5ʹ-ACGGATGTCAACGTCACACT-3ʹ).

### 2.7 ELISA

The concentrations of inflammatory mediators and cytokines in the culture supernatants of BV2 cells were assessed using ELISA kits for TNF-α (#MTA00B R&D Systems, Minneapolis, MN), IL-1β (#MLB00C R&D Systems, Minneapolis, MN), and PGE_2_ (#KGE004B R&D Systems, Minneapolis, MN) according to the manufacturer’s instructions.

### 2.8 Western blot analysis

Proteins were isolated from BV2 cells using radio-immunoprecipitation assay (RIPA) buffer containing protease and phosphatase inhibitors (Roche Diagnostics, Germany), followed by western blot analysis [16]. The primary antibodies against COX-2 (#ab179800), Iba1 (#178846), TREM2 (#209814), and secondary antibodies (#ab150077) were obtained from Abcam (Cambridge, MA, USA). The bands were analyzed using the Image J software [16].

### 2.9 Statistical analysis

Statistical analyses were performed using the GraphPad Prism statistical software. Data were analyzed with Analysis of Variance (ANOVA) and shown as mean ± standard error of the mean (S.E.M.). *P* < 0.05 was considered statistically significant.

## 3. Results

Using an *in vitro* BV2 microglial cell model stimulated with isoflurane, we investigated the benefits of maprotiline on oxidative stress, expression of inflammatory factors, and the TREM2 signaling pathway.

### 3.1 The effects of maprotiline on BV2 cells viability

**Figure 1.**
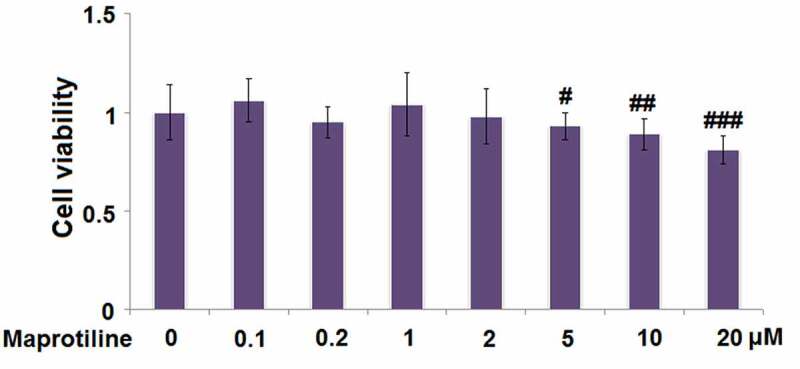
The effects of Maprotiline on cell viability in BV2 microglial cells. (a). Molecular structure of Maprotiline; (b). Cells were stimulated with Maprotiline at the concentrations of 0, 0.1, 0.2, 1, 2, 5, 10, 20 μM for 24 hours. Cell viability was measured using WST-1 assay (#, ##, ###, P < 0.05, 0.01, 0.005 vs. vehicle group)

Before the experiments, we first tested whether maprotiline (molecular structure is shown in Figure 1(a)) has cytotoxicity toward BV2 cells. BV2 cells were stimulated with the vehicle (PBS) or maprotiline (0.1, 0.2, 1, 2, 5, 10, 20 μM) for 24 h, and then WST-1 assay was conducted. The results show that maprotiline did not present toxicity at lower concentrations of 0.1, 0.2, 1, and 2 μM (Figure 1(b)). Therefore, 1 and 2 μM were used in the following experiments.

### 3.2 Maprotiline mitigated isoflurane-induced cell injury in BV2 cells

**Figure 2.**
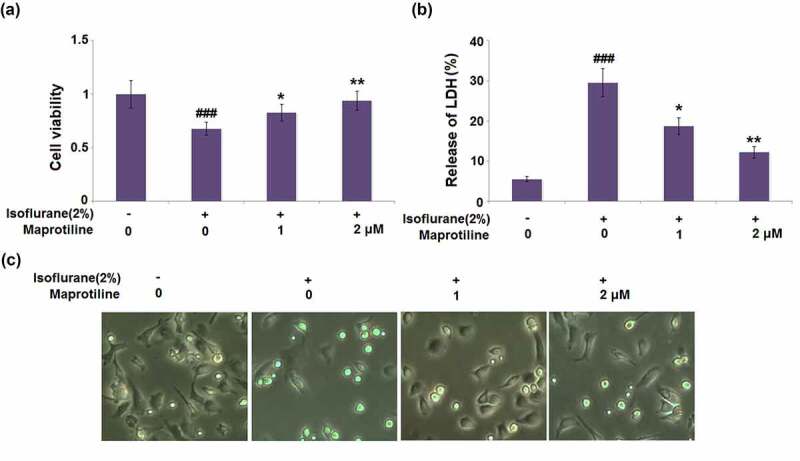
Maprotiline ameliorated Isoflurane-induced reduction of cell viability and release of LDH in BV2 microglial cells. Cells were stimulated with 2% Isoflurane with or without Maprotiline (1, 2 μM) for 24 hours. (a). Cell viability; (b). Release of LDH; (c). Cell morphology of BV2 microglial cells (###, P < 0.005 vs. vehicle group; *, **, P < 0.05, 0.01 vs. Isoflurane group)

BV2 cells were pretreated with maprotiline (1 and 2 μM) for 2 h, followed by stimulation with 2% isoflurane for 24 h [17]. Compared to the vehicle-treated BV2 cells (1 ± 0.13), isoflurane-induced BV2 cells displayed significantly reduced cell viability (0.68 ± 0.06). Pretreatment with maprotiline (1 and 2 μM) followed by isoflurane-stimulation improved the cell viability to 0.83 ± 0.08 and 0.94 ± 0.09, respectively (Figure 2(a)). Isoflurane significantly increased the release of LDH (29.6%±3.52%), as compared to the vehicle-treated BV2 cells (5.6%±0.65%). However, maprotiline (1 and 2 μM) significantly reduced the LDH release to 18.8%±2.1% and 12.3%±1.4% (Figure 2(b)). The morphology of BV2 microglial cells under different conditions is shown in Figure 2(c). These results show that maprotiline mitigated isoflurane-induced cell injury.

### 3.3 Maprotiline ameliorated isoflurane-induced oxidative stress in BV2 cells

**Figure 3.**
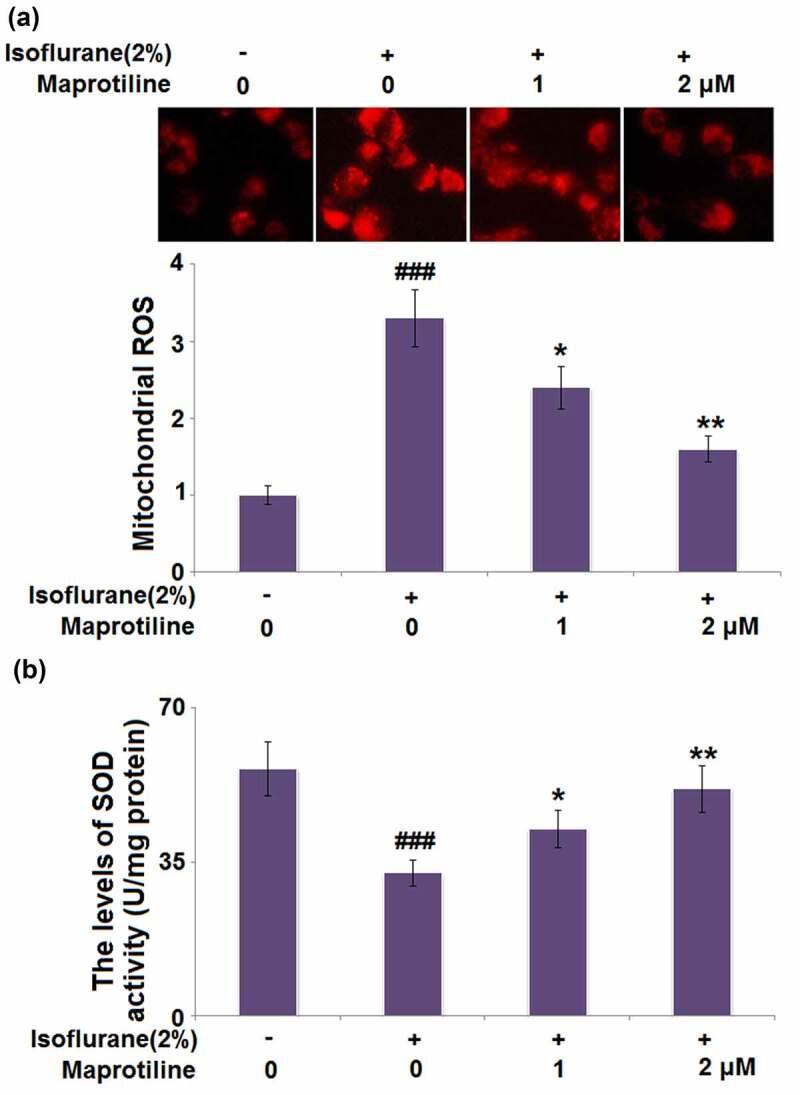
Maprotiline ameliorated Isoflurane-induced oxidative stress in BV2 microglial cells. (a). Mitochondrial ROS; (b). The levels of SOD activity (###, P < 0.005 vs. vehicle group; *, **, P < 0.05, 0.01 vs. Isoflurane group)

We then investigated if maprotiline treatment could alter isoflurane-induced oxidative stress. Figure 3(a) shows that stimulation with 2% isoflurane increased the level of mitochondrial ROS (3.3 ± 0.37) compared with the vehicle treatment without isoflurane (1 ± 0.12). Mitochondrial ROS levels were significantly reduced to 2.4 ± 0.27 and 1.6 ± 0.17 after 24 h incubation with maprotiline (1 and 2 μM). When BV2 cells were incubated with 2% isoflurane, the level of SOD activity was reduced to 32.5 ± 2.96 U/mg, as compared to the vehicle-treated BV2 cells (56.3 ± 6.21 U/mg). By contrast, maprotiline (1 and 2 μM) treatment upregulated the decreased SOD activity to 42.6 ± 4.15 and 51.7 ± 5.38 U/mg, respectively (Figure 3(b)). The data indicate that maprotiline could suppress oxidative stress via inhibiting the generation of ROS and rescuing SOD activity.

### 3.4 Maprotiline suppressed isoflurane-induced the expression of TNF-α and IL- 1β

**Figure 4.**
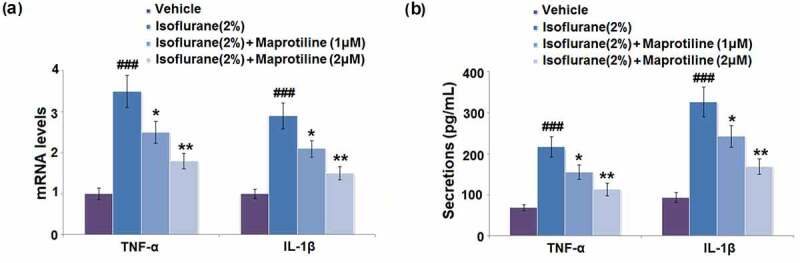
Maprotiline suppressed Isoflurane-induced expression of pro-inflammatory cytokines. (a). mRNA levels of TNF-α and IL-1β; (b). Secretions of TNF-α and IL-1β (###, P < 0.005 vs. vehicle group; *, **, P < 0.05, 0.01 vs. Isoflurane group)

To explore the regulatory effect of maprotiline on isoflurane-induced inflammatory response in BV2 cells, expression levels of TNF-α and IL-1β were measured with RT-PCR and ELISA. Isoflurane exposure caused a significant increase in the mRNA levels of TNF-α and IL-1β by 3.5- and 2.9-fold, compared to the vehicle-treatment group (Figure 4(a,b)). However, maprotiline (1 and 2 μM) pretreatment significantly decreased the mRNA levels of TNF-α by 28.6% and 48.6% and decreased the mRNA levels of IL-1β by 27.6% and 48.3% (Figure 4(a)). The secretion levels of TNF-α and IL-1β were increased from 68.5 ± 7.25 pg/mL and 93.8 ± 11.5 pg/mL to 217.3 ± 25.38 pg/mL and 326.7 ± 36.56 pg/mL after isoflurane-treatment in BV2 cells. Moreover, pretreatment of BV2 cells with maprotiline (1 μM) decreased the secretion levels of TNF-α and IL-1β to 155.2 ± 17.52 pg/mL and 242.6 ± 26.82 pg/mL, while maprotiline (2 μM) decreased the secretion levels of TNF-α and IL-1β to 113.8 ± 15.32 pg/mL and 168.9 ± 18.96 pg/mL (Figure 4(b)). These results show that maprotiline suppressed isoflurane-induced expression of pro-inflammatory cytokines at both mRNA and protein levels.

### 3.5 Maprotiline inhibited the expression of COX-2 and the production of PGE_2_

**Figure 5.**
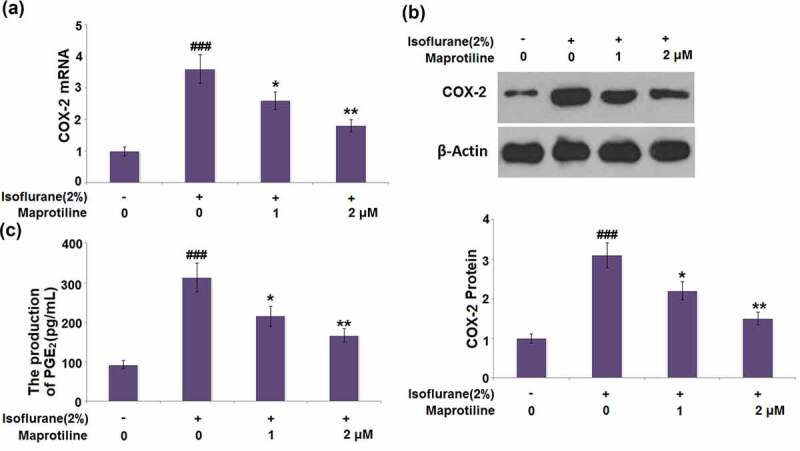
Maprotiline inhibited the expression of COX-2 and the production of PGE_2_. Cells were stimulated with 2% Isoflurane with or without Maprotiline (1, 2 μM) for 24 hours. (a). mRNA levels of COX-2; (b). Protein levels of COX-2; (c). The production of PGE_2_ (###, P < 0.005 vs. vehicle group; *, **, P < 0.05, 0.01 vs. Isoflurane group)

We then examined the effect of maprotiline on the two-inflammatory mediators, COX-2 and PGE_2_. Isoflurane significantly increased the mRNA and protein levels of COX-2 by 3.6 and 3.1 folds (Figure 5(a,b)). On the other hand, the mRNA and protein levels of COX-2 were markedly decreased in the presence of maprotiline (1 and 2 μM). Isoflurane-treatment significantly induced the production of PGE_2_ by 2.4-fold. By contrast, maprotiline (1 and 2 μM) downregulated the isoflurane-mediated increase in the PGE_2_ production by 0.32- and 0.47- fold, respectively (Figure 5(c)). These results demonstrate the anti-inflammatory effect of maprotiline in isoflurane-treated BV2 microglial cells.

### 3.6 Maprotiline inhibited the expression of Iba1 in BV2 cells

**Figure 6.**
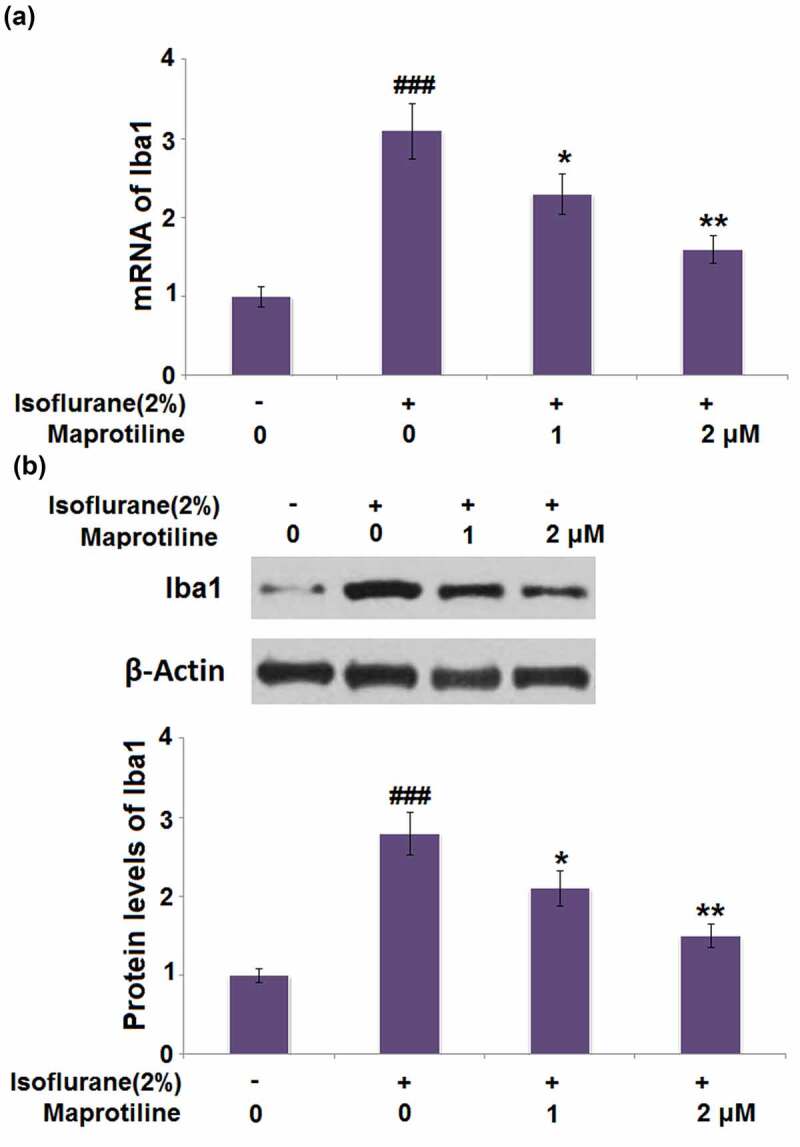
Maprotiline inhibited the expression of Iba1 in BV2 microglial cells. (a). mRNA of Iba1; (b). Protein levels of Iba1 (###, P < 0.005 vs. vehicle group; *, **, P < 0.05, 0.01 vs. Isoflurane group)

ba1 is a marker of microglia and indicates their activation. The mRNA of Iba1 reached a 3.1-fold increase in cells stimulated with isoflurane. A 25.8% and 48.4% reduction of Iba1 mRNA was observed in maprotiline (1 and 2 μM) treatment groups (Figure 6(a)). Meanwhile, the protein level of Iba1 was increased by 2.8-fold in isoflurane-stimulated cells but decreased by 0.25-and 0.46-fold in maprotiline (1 and 2 μM)-treated cells (Figure 6(b)). These results indicate that maprotiline inhibited isoflurane-induced activation of BV2 cells.

### 3.7 Isoflurane reduced the expression of TREM2 in BV2 microglia cells

**Figure 7.**
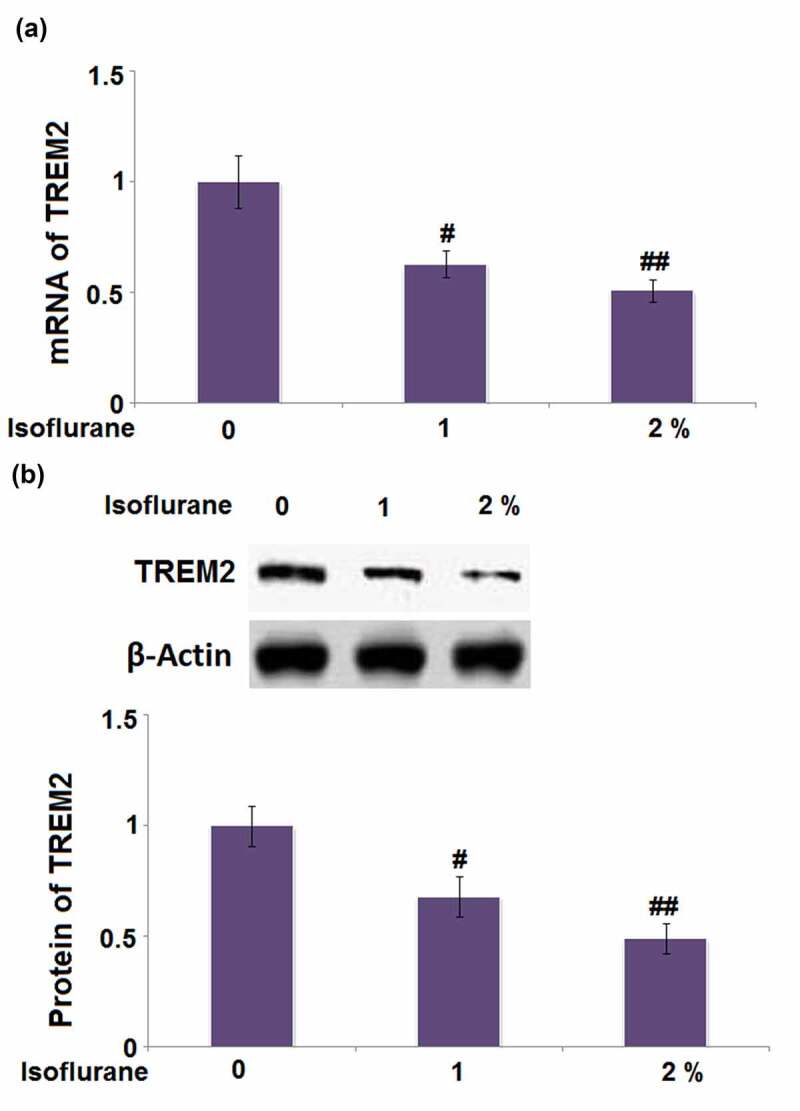
Isoflurane reduced the expression of TREM2 in BV2 microglia cells. Cells were stimulated with 1% and 2% Isoflurane for 24 hours. (a). mRNA of TREM2; (b). Protein of TREM2 (#, ##, P < 0.05, 0.01 vs. vehicle group)

TREM2 is one of the microglial receptors and is crucial for the regulation of inflammatory response. To test whether TREM2 is involved in the isoflurane-induced inflammatory response, its expression was quantified. As shown in Figure 7a, cells stimulated with 1% and 2% isoflurane respectively exhibited a 0.37- and 0.49-fold decrease in the mRNA level of TREM2. The protein level of TREM2 in cells stimulated with 1% and 2% isoflurane was reduced by 0.32- and 0.51-fold, respectively (Figure 7(b)). These findings display that TREM2 might be involved in the isoflurane-induced inflammatory response in BV2 microglial cells.

### 3.8 Maprotiline restored isoflurane-induced reduction of TREM2 in BV2 microglial cells

**Figure 8.**
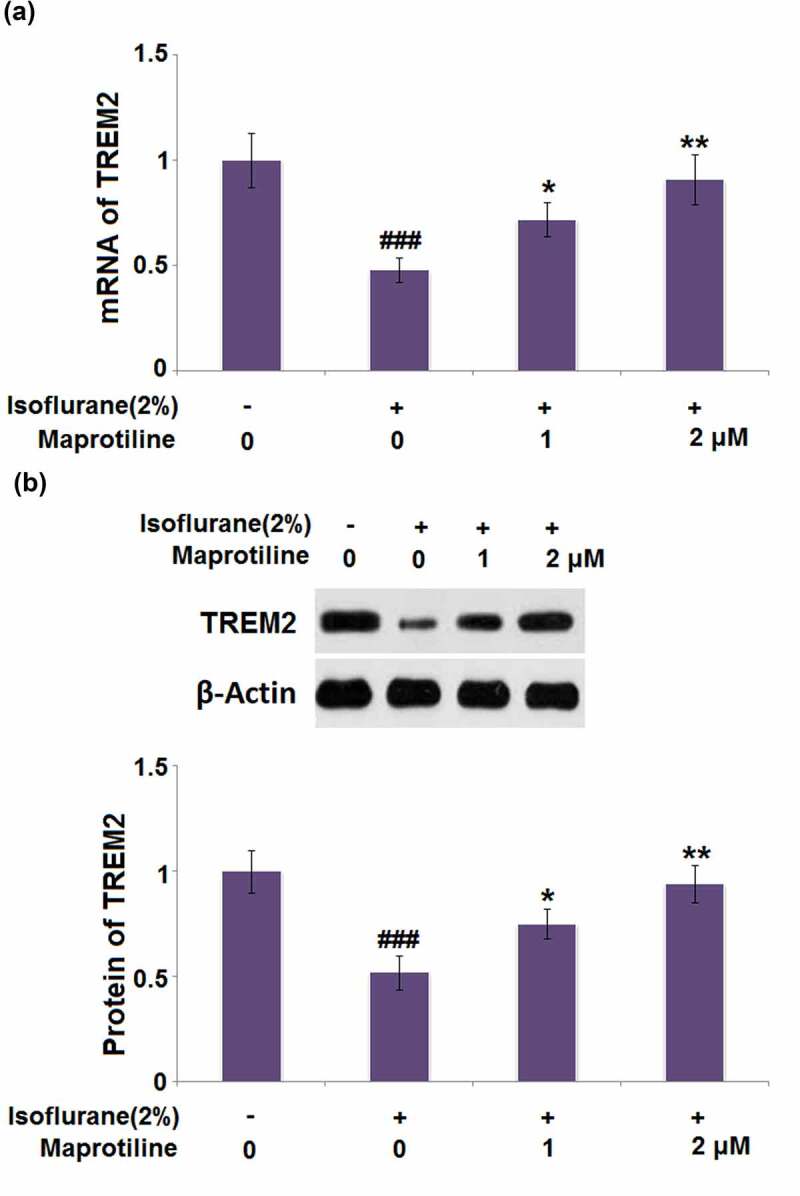
Maprotiline restored Isoflurane-induced reduction of TREM2 in BV2 microglia cells. Cells were stimulated with 2% Isoflurane with or without Maprotiline (1, 2 μM) for 24 hours. (a). mRNA of TREM2; (b). Protein of TREM2 (###, P < 0.005 vs. vehicle group; *, **, P < 0.05, 0.01 vs. Isoflurane group)

We studied the effect of maprotiline on the expression of TREM2 in isoflurane-challenged BV2 cells. The mRNA level of TREM2 was restored by maprotiline (1 and 2 μM) with a 1.5- and 1.9-fold change (Figure 8(a)). The protein level of TREM2 was increased in maprotiline (1 and 2 μM) treated cells by 1.4- and 1.8-fold, respectively (Figure 8(b)).

### 3.9 The beneficial effects of maprotiline against isoflurane are mediated by TREM2

**Figure 9.**
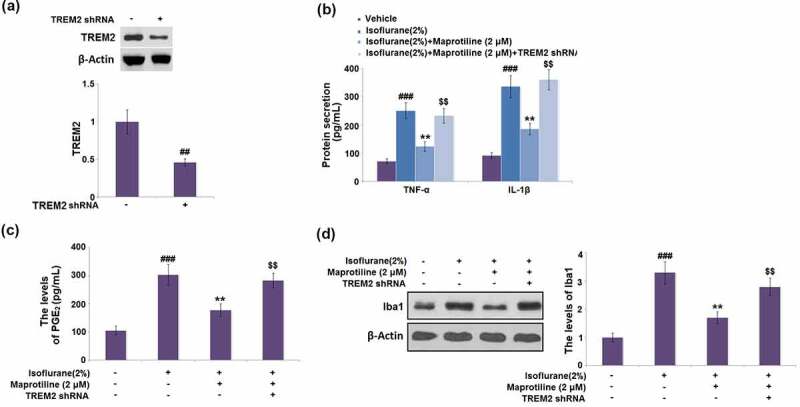
The beneficial effects of Maprotiline against Isoflurane are mediated by TREM2. Cells were transduced with lentiviral TREM2 shRNA, followed by stimulation with 2% Isoflurane with or without Maprotiline (2 μM) for 24 hours. (a). Western blot results revealed successful knockdown of TREM2; (b). The levels of TNF-α, IL-1β; (c). The levels of PGE_2_; (d). The levels of Iba1 (###, P < 0.005 vs. vehicle group; **, P < 0.01 vs. Isoflurane group; $$, Isoflurane+ Maprotiline group)

To further confirm the role of TREM2, BV2 cells were transduced with LV-shTREM2. Compared with the vehicle control, the protein level of TREM2 was markedly reduced by 54% in cells transduced with LV-shTREM2 (Figure 9(a)). The maprotiline-caused decreases in the levels of TNF-α, IL-1β, and PGE_2_ were elevated by 1.9-, 1.9- and 1.6-fold respectively in LV-shTREM2- transduced cells (Figure 9(b,c)). Western blot analysis showed that the protein level of Iba1 was increased by 1.6-fold in LV-shTREM2-transduced cells (Figure 9(d)). These results suggest that the beneficial effects of maprotiline against isoflurane are mediated by TREM2.

## 4. Discussion

Microglia are a type of innate immune cells and serve as resident phagocytes in the central nervous system (CNS). Microglia play crucial roles in maintaining the homeostasis of the CNS tissue and contribute to the development of brain tissue [18]. Microglia also play an important role in neuronal injury response and pathogen defense. In response to insult, microglia may be activated, and thereby secrete pro-inflammatory and anti-inflammatory neuroprotective factors that cause neuroinflammation and enhance cytotoxicity [19]. Excessive activation of microglia leads to the death of neurons and damages the neural tissue, which in turn exacerbates the microglial activation and causes progressive loss of neurons [18]. Hence, resolving the microglial activation-mediated neuroinflammation and microglial injury represents a novel treatment strategy for neuronal damage.

The common anesthetic isoflurane causes obvious increases in the levels of proinflammatory cytokines, TNF-α, IL-6, and IL-1β, in both mice brain tissues and primary neurons, which may lead to neuroinflammation [20]. In a male newborn piglet, exposure to isoflurane results in the activation of microglia and causes significant alternations to the genes mediating memory formation and recall [21]. Isoflurane induces cognitive impairment of aged mice and induces inflammation and oxidative stress in BV2 cells through the ROS-p38MAPK/ATF2 pathway [22]. Isoflurane induces the increased protein levels of TNF-α, IL-1β, interferon (IFN)-γ, and microglia marker Iba-1 [23]. Isoflurane increases the expressions of IL-1β, TNF-α, and IL-6, and causes polarization of BV2 microglial cells via the TLR4 signaling pathway [24]. These data indicate that isoflurane induces microglial activation and mediates neuroinflammation. Here, we found that isoflurane caused microglial activation, as evidenced by the decreased cell viability, increased LDH release, increased production of inflammatory markers and oxidative indicators, as well as the increased expression of Iba1. The effects of isoflurane exposure on oxidative stress, inflammation, and cell damage in BV2 cells were attenuated by maprotiline treatment.

TREM2 is expressed by microglia and has been identified as an essential immune receptor [25]. TREM2 plays an active role in the pathogenesis of various neurodegenerative diseases (NDDs) [26]. Emerging evidence indicates that TREM2 is implicated in various cellular processes of microglia, such as proliferation, survival, and phagocytic activity [27]. Furthermore, TREM2 has the ability to enhance microglial phagocytosis and suppress inflammation. Zhang *et al*. [27] reported that the knockdown of TREM2 modulates microglia phenotypes and leads to the exaggeration of inflammatory responses in BV2 cells, while TREM2 overexpression alleviates the inflammation. TREM2 overexpression represses LPS-induced inflammatory responses via the inhibition of PI3K/NF-κB signaling in BV2 cells [28]. TREM2- knockout mice showed a decreased transcription of the pro-inflammatory cytokines TNF-α, IL-1α, and IL-1β, associated with reduced microglial activity (CD68, Iba1) [29]. Moreover, Lue *et al*. demonstrated that TREM2 upregulated the expression of Iba1 in patients with Alzheimer’s Disease [30]. This finding showed the relationship between TREM2 and microglial activation. Hence, we examined whether TREM2 is involved in the protective effect of maprotiline in BV2 microglial cells. The results display that maprotiline treatment restored the isoflurane-induced reduction of TREM2 in BV2 microglial cells. Moreover, the knockdown of TREM2 abolished the beneficial effects of maprotiline against isoflurane, which indicates that the protective effect of maprotiline was mediated by TREM2.

With the protective potency and multiple-targeting capacity of maprotiline, it is speculated that maprotiline pre-conditioning conferred significant neuroprotection against isoflurane-induced brain injury by anti-microglial inflammation mechanisms. These results advance our understanding of the possibility that maprotiline might be used as a clinical agent to neutralize isoflurane-induced neuroinflammation and cognitive dysfunction. Nevertheless, further assessment to verify the function of maprotiline is necessary to draw a firm conclusion.

However, a major limitation of our study is that we only examined the protective effects of maprotiline against isoflurane-induced microglial cell activation in an *in vitro* BV2 cell model. Indeed, the molecular mechanism of microglial activation in the brain is complicated and needs to be elucidated. Therefore, current preclinical research is largely dependent on animal models. A future study with ideal animal models of microglial activation will provide a more complete picture.

## Conclusion

In this study, we bolstered our speculation of maprotiline in isoflurane-induced BV2 microglial cells. Maprotiline exerted a protective effect against isoflurane-caused microglial activation via increasing the expression of TREM2. These findings might help to develop an optimal therapeutic strategy for preventing isoflurane-caused neurotoxicity.

## Data Availability

Data of this study is/are available upon reasonable request to the corresponding authors.
